# Supplementary material for: Total and specific immunoglobulin E in induced sputum in allergic and non-allergic asthma
Source: PLoS One. 2020 Jan 29;15(1):e0228045. doi: 10.1371/journal.pone.0228045 (PMC6988954; doi:10.1371/journal.pone.0228045)
Supplement: Attachment — Submitted filename: Dear Editor.docx [file pone.0228045.s001.docx]

Dear Editor,

The article entitled as “Total and specific immunoglobulin E in induced sputum in allergic and non-allergic Asthma” was reviewed for PLOS ONE on 20 November 2019. It is a valuable study in means of using and evaluating induced sputum and it has a control group. However there are the following points that needs to be solved;

- The size of the study group seems to be too small, this may be increased, or the power of the study may be calculated and added.
- The subject of the study that comparing total IgE between allergic and nonallergic asthma is not new even tough using induced sputum and spesicifc IgE.
- Local allergy or entopy was showed in nonallergic rhinitis or asthma in mucosa biopsy or lavage in 2000’s. However, it has lost its popularity because of the difficulty to diagnose these patients, and not changing the treatment. Furthermore, it is not evaluated as an asthma phenotype. The new point may be investigating entopy between allergic, non-allergic eosinophilic, and non-allergic non-eosinophilic phenotypes.
- Subgroup analysis as high and low total ıge may be performed.
- There are other allergens such as pollens that may be present in nonallergic asthmatics sputum. This question effects the results unconfident.
- Mold sensitivity has a great impact on total IgE, and should have been explored.
