# Supplementary material for: Total and specific immunoglobulin E in induced sputum in allergic and non-allergic asthma
Source: PLoS One. 2020 Jan 29;15(1):e0228045. doi: 10.1371/journal.pone.0228045 (PMC6988954; doi:10.1371/journal.pone.0228045)
Supplement: Attachment — Submitted filename: Response to Reviewers.docx [file pone.0228045.s002.docx]

**Response to Editor and Reviewers**

1. When submitting your revision, we need you to address these additional requirements.
Please ensure that your manuscript meets PLOS ONE's style requirements, including those for file naming. The PLOS ONE style templates can be found at
http://www.journals.plos.org/plosone/s/file?id=wjVg/PLOSOne_formatting_sample_main_body.pdf and <http://www.journals.plos.org/plosone/s/file?id=ba62/PLOSOne_formatting_sample_title_authors_affiliations.pdf>

***Author’s response:*** *Thank you for directing us to the right places to improve the style requirements. The necessary changes have been made.*

2. We noticed you have some minor occurrence(s) of overlapping text with the following previous publication(s), which needs to be addressed:
https://doi.org/10.1159/000431259
https://doi.org/10.2147/JAA.S142200
http://www.jiaci.org/issues/vol25issue6/6.pdf

In your revision ensure you cite all your sources (including your own works), and quote or rephrase any duplicated text outside the Methods section. Further consideration is dependent on these concerns being addressed.

***Author’s response:*** *We are very sorry for the coincidence with the publications you mention. In all three cases they are articles written by one of the authors of the current article, and we have detected similar phrases in the part of methods that have been modified.*

3. We note that you have reported significance probabilities of 0 in places. Since p=0 is not strictly possible, please correct this to a more appropriate limit, eg 'p<0.0001'.

***Author’s response:*** *Thanks for the commentary; the change has been made both in the text and in the tables.*

4. We note that you have indicated that data from this study are available upon request. PLOS only allows data to be available upon request if there are legal or ethical restrictions on sharing data publicly. For information on unacceptable data access restrictions, please see http://journals.plos.org/plosone/s/data-availability#loc-unacceptable-data-access-restrictions.
In your revised cover letter, please address the following prompts:
a) If there are ethical or legal restrictions on sharing a de-identified data set, please explain them in detail (e.g., data contain potentially identifying or sensitive patient information) and who has imposed them (e.g., an ethics committee). Please also provide contact information for a data access committee, ethics committee, or other institutional body to which data requests may be sent.
b) If there are no restrictions, please upload the minimal anonymized data set necessary to replicate your study findings as either Supporting Information files or to a stable, public repository and provide us with the relevant URLs, DOIs, or accession numbers. Please see http://www.bmj.com/content/340/bmj.c181.long for guidelines on how to de-identify and prepare clinical data for publication. For a list of acceptable repositories, please see http://journals.plos.org/plosone/s/data-availability#loc-recommended-repositories.
We will update your Data Availability statement on your behalf to reflect the information you provide.

***Author’s response:*** *Thanks to your kind recommendations, we contacted our ethics committee and were informed of the freedom to share the database. It is currently available for consultation at* [www.figshare.com](http://www.figshare.com) *(DOI 10.6084/m9.figshare.11499162.v1)*

5. Your ethics statement must appear in the Methods section of your manuscript. If your ethics statement is written in any section besides the Methods, please move it to the Methods section and delete it from any other section. Please also ensure that your ethics statement is included in your manuscript, as the ethics section of your online submission will not be published alongside your manuscript.

***Author’s response:*** *As requested, the ethics statement has been moved to the “methods” section.*

6. Thank you for stating the following in the Competing Interests section:

**AC** in the last three years received honoraria for speaking at sponsored meetings from AstraZeneca, Chiesi, Esteve Laboratories, Faes Farma, Ferrer, GlaxoSmithKline, Novartis, Teva, Zambon.  Received help assistance to meeting travel from Bial, Novartis. Act as a consultant for AstraZeneca, Boehringer, GlaxoSmithKline, Novartis. And received funding/grant support for research projects from a variety of Government agencies and not-for-profit foundations, as well as AstraZeneca. **EC** has received funding to travel to and attend training activities from ALK, Menarini, Teva, AstraZeneca, Chiesi, Boehringer, and Novartis. **LS, EM** and **AG** declare no conflict of interests. **JG** has received funding to travel and attend to training activities from Menarini, Teva, AstraZeneca, Chiesi, GSK, Mundipharma, Boehringer. In the last three years, **VP** has received honoraria for speaking at sponsored meetings from AstraZeneca, Boehringer-Ingelheim, Chiesi, GSK, and Novartis. VP has also received financial support to travel to meetings organized by Chiesi and Novartis. VP is a consultant for ALK, AstraZeneca, Boehringer, MundiPharma, and Sanofi. VP has also received funding/grant support for research projects from a variety of governmental agencies and not-for-profit foundations, as well as from AstraZeneca, Chiesi and Menarini.

Please confirm that this does not alter your adherence to all PLOS ONE policies on sharing data and materials, by including the following statement: "This does not alter our adherence to  PLOS ONE policies on sharing data and materials.” (as detailed online in our guide for authors [http://journals.plos.org/plosone/s/competing-interests](https://excas01.dspau.santpau.es/owa/redir.aspx?C=lYmgd4lgNuIcj90kzfkut6xgpMJ2gK7KtVtjhSx3yuKDCzZbTZDXCA..&URL=http%3a%2f%2fjournals.plos.org%2fplosone%2fs%2fcompeting-interests)).  If there are restrictions on sharing of data and/or materials, please state these. Please note that we cannot proceed with consideration of your article until this information has been declared.

Please include your updated Competing Interests statement in your cover letter; we will change the online submission form on your behalf.

Please know it is PLOS ONE policy for corresponding authors to declare, on behalf of all authors, all potential competing interests for the purposes of transparency. PLOS defines a competing interest as anything that interferes with, or could reasonably be perceived as interfering with, the full and objective presentation, peer review, editorial decision-making, or publication of research or non-research articles submitted to one of the journals. Competing interests can be financial or non-financial, professional, or personal. Competing interests can arise in relationship to an organization or another person. Please follow this link to our website for more details on competing interests: [http://journals.plos.org/plosone/s/competing-interests](https://excas01.dspau.santpau.es/owa/redir.aspx?C=lYmgd4lgNuIcj90kzfkut6xgpMJ2gK7KtVtjhSx3yuKDCzZbTZDXCA..&URL=http%3a%2f%2fjournals.plos.org%2fplosone%2fs%2fcompeting-interests)

***Author’s response:*** *As you kindly suggested, the competing interests have been modified and added at the end of the cover letter, to be changed at the online submission. The authors' declarations have no bearing on the adherence to PLOS ONE policies on sharing data and materials, and therefore, the suggested statement has been added at the end.*

**Comments to Reviewers:**

**1. Is the manuscript technically sound, and do the data support the conclusions?**

The manuscript must describe a technically sound piece of scientific research with data that supports the conclusions. Experiments must have been conducted rigorously, with appropriate controls, replication, and sample sizes. The conclusions must be drawn appropriately based on the data presented.

Reviewer #1: Partly

Reviewer #2: No

***Author’s response:*** *According to the recommendations of the reviewer, we have better drafted the manuscript in key points to make it more understandable, especially in the conclusions (see line 41)*

**2. Has the statistical analysis been performed appropriately and rigorously?**

Reviewer #1: N/A

Reviewer #2: No

***Author’s response:*** *The statistical analysis was carried out under the support of a statistician who guided us at all times on the most appropriate analyzes for this study. If you suggest doing some analysis of greater interest in our study, using the database that we have attached to the study, we would be delighted to perform it.*

**3. Have the authors made all data underlying the findings in their manuscript fully available?**

The PLOS Data policy requires authors to make all data underlying the findings described in their manuscript fully available without restriction, with rare exception (please refer to the Data Availability Statement in the manuscript PDF file). The data should be provided as part of the manuscript or its supporting information, or deposited to a public repository. For example, in addition to summary statistics, the data points behind means, medians and variance measures should be available. If there are restrictions on publicly sharing data—e.g. participant privacy or use of data from a third party—those must be specified.

Reviewer #1: Yes

Reviewer #2: Yes

***Author’s response:*** *As required by the internal policy of Plos one, we have attached our data to a public repository.*

**4. Is the manuscript presented in an intelligible fashion and written in standard English?**

PLOS ONE does not copyedit accepted manuscripts, so the language in submitted articles must be clear, correct, and unambiguous. Any typographical or grammatical errors should be corrected at revision, so please note any specific errors here.

Reviewer #1: Yes

Reviewer #2: Yes

***Author’s response:*** *Thanks for your feedback*

**5. Review Comments to the Author**

Please use the space provided to explain your answers to the questions above. You may also include additional comments for the author, including concerns about dual publication, research ethics, or publication ethics. (Please upload your review as an attachment if it exceeds 20,000 characters)

**Reviewer #1: Dear Editor,**

The article entitled as “Total and specific immunoglobulin E in induced sputum in allergic and non-allergic Asthma” was reviewed for PLOS ONE on 20 November 2019. It is a valuable study in means of using and evaluating induced sputum and it has a control group. However there are the following points that needs to be solved;

- The size of the study group seems to be too small, this may be increased, or the power of the study may be calculated and added.

***Author’s response:*** *Due to the limited literature and great variability in the results that exist of the IgE values in the induced sputum, for the calculation of the sample size, the reference values of the IgE in the induced sputum will be considered as binding variable reports in literature (references 31 and 32). This sample size is calculated with the Granmo V7.10 program, setting the type I error at the usual 5% (α = 0.05), with a bilateral approximation and with a minimum difference required for a power of 80% or higher (ß = 0.2). Also our study had as a secondary objective to establish a preliminary estimate of total IgE and Der p 1-specific IgE in the induced sputum of healthy individuals. To support the results of this work, a larger sample size would be necessary for future studies. A comment has been added in the manuscript (see line 327)*

- The subject of the study that comparing total IgE between allergic and nonallergic asthma is not new even tough using induced sputum and specific IgE.

***Author’s response****:* The levels of total and specific IgE in blood are extensively studied comparatively in asthmatic and healthy patients, and also in different types of asthmatic patients. However, sputum-induced levels of this immunoglobulin have been poorly reported. Our particular interest was to assess its potential applicability as a diagnostic test in the detection of local allergic reactions at the bronchial level.

- Local allergy or entopy was showed in nonallergic rhinitis or asthma in mucosa biopsy or lavage in 2000’s. However, it has lost its popularity because of the difficulty to diagnose these patients, and not changing the treatment. Furthermore, it is not evaluated as an asthma phenotype. The new point may be investigating entopy between allergic, non-allergic eosinophilic, and non-allergic non-eosinophilic phenotypes.

***Author’s response:*** We totally agree with the reviewer's opinion. Local allergy has been a controversial issue in the last decade, and in our view further research is needed to define this entity properly. Its loss of popularity has been due, on the one hand, to diagnostic difficulty, so this study has been, in fact, a first approach to a technique that could allow diagnosing it in a simple way. On the other hand, until now it did not imply differences in treatment, but we believe that with the availability of new monoclonal drugs this may change. We also consider that it could help to better understand a group of patients, who because a priori they do not present signs of atopy or eosinophilia, remain limited to conventional treatments.

- Subgroup analysis as high and low total IgE may be performed.

***Author’s response:*** The main focus of our work was:

1) To measure the levels of total and Dermatophagoides pteronyssinus (d1) specific IgE in the induced sputum (IS) of asthmatic patients and healthy volunteers

2) To correlate the levels of total local IgE and specific IgE to d1 (sputum and peripheral blood) in patients with allergic and non-allergic asthma

Since there are few published papers in the literature that have measured levels of total IgE in sputum and since there are no established values of normal sputum total IgE levels, we cannot make this classification as there is no established cut-off point.

- There are other allergens such as pollens that may be present in nonallergic asthmatics sputum. This question effects the results unconfident.

***Author’s response****:* Our inclusion criteria were particularly strict and detailed in this study. We only selected patients with allergic asthma in whom *D. pteronyssinus* was the only clinically relevant allergen, in addition to presenting positive prick test, and/or previous high specific IgE for this allergen. If they presented a positive prick test for other allergens and there was any suspicion that their symptoms were related to other allergens to which they were sensitized, they were excluded from the study. This has been clarified in the manuscript (see line 140)

- Mold sensitivity has a great impact on total IgE, and should have been explored.

***Author’s response:*** This is an interesting point of view, but in our area, dust mites are the most common perennial allergens, and mold sensitization is not particularly common. In fact, in this group of patients, only one of them had a positive prick test for Aspergillus, and it was not clinically relevant (see line 219)

Reviewer #2: The detection limit was 0.35 kU/L for specific IgE and yet the authors report levels in sputum markedly below this level, e.g. for Der p-specific IgE 0.055 kU/L median and 0.04 kU/L average for non-allergic asthma. (See line 173-4 and Table 2.) Thus unfortunately, the data appear to be invalid.

***Author’s response:*** According to manufacturer’s recommendation, > 2kU/L for total IgE and > 0.35kU/L for specific IgE, are the values to consider a positive test, but as we previously stated, the levels in induced sputum are not described yet. We understand that the previous phrasing could be confusing, so we have modified the corresponding paragraph to better understand this aspect.
